# Medical Defence or Protection Societies

**Published:** 1907-04-27

**Authors:** 


### Medical Defence or Protection Societies.

The responsibilities involved in medical practice are so numerous and touch so many interests, that, at times, questions must almost inevitably arise which involve legal controversies and disputes. Such disputes may occur either between different members of the profession, or between a medical practitioner and some private individual or public body. In either case, the persons immediately interested will certainly need advice, based alike upon common sense and technical knowledge. It is therefore fortunate, from the point of view of the medical profession, that organisations have come into existence capable of meeting these wants. These organisations are founded upon the principles of mutual insurance and co-operation, so that the risks of the individual are spread over a relatively large membership. Hence, for a small annual subscription, any practitioner may have at his disposal, in time of need, the moral and material support of an experienced organisation. Judging, however, from existing facts, it would seem that many members of the profession fail to appreciate these advantages, with the result that they leave themselves unprotected from certain manifest risks, while, at the same time, the organisations in question are not so strong and influential as would otherwise be the case. It seems so obvious that in disputes involving medical questions the practitioner immediately interested would be immensely helped by association with his fellows, that we venture to suggest to any of our readers who have hitherto failed to do so, the advisability of at once becoming a member of one or other of these medical defence or protection societies. Both the London and Counties Medical Protection Society, and the Medical Defence Union, enjoy the highest professional sanction, and are conducted, under such sanction, by capable and experienced officers. It is perhaps to be regretted that there should be any rivalry in this field. But with this we are not concerned. Our immediate regard is the interest of the individual practitioner, and our claim is that this needs the help and advantages that can be secured by membership of one or other of these organisations.

There is no need to recite the special legal risks which are attached to medical practice. Charges of malpractice, attempts at blackmail, and the like, find their way every now and then into the public prints, and thus, apart altogether from direct experience, every practitioner ought to be aware of the dangers to which he is exposed. But to trust to chance is so large an element in human nature, and it is so easy to believe that misfortune and difficulty are for others rather than for oneself, that many take the risk rather than provide for the chance of its active development. Hence it repeatedly happens that a practitioner finds himself involved in a serious legal dispute without the assistance and support which the payment of a small annual subscription would have secured ready to his hand. He may be, and indeed in such circumstances is usually proved to be, entirely free from legal and moral blame. Yet he has to pay a severe penalty in the shape of natural anxiety and often, also, in the shape of a bill of costs, from which he would have been saved, at least in a large measure, had he only in due time exercised a proper degree of prescience. It is true that in circumstances such as those just alluded to the profession often comes to the rescue with a subscription list. But this cannot be counted on, and even when it occurs the practitioner concerned occupies a much less dignified position than that which would have been his due had he merely received corporate assistance as a right secured to himself on the basis of insurance and mutual protection. Again, short of serious legal disputes involving a trial in open court, there are many circumstances in which the practitioner may gain great help from membership of one or other of the defence organisations. Difficulties with fellow practitioners, with individual patients, or with public bodies, are often allowed to grow into dangerous proportions for want of a little tact in their early stages. And here, advice from those who have almost daily experience of such difficulties is of immense value. Further, to attack an individual practitioner seems to a certain type of person a comparatively simple and secure proceeding, but the matter takes on a very different complexion when it is known that the attack will be met with all the resources and prestige of a powerful and experienced organisation. Membership of such a society, therefore, not only secures financial and material support; in adition, it is a protection against unjust demands and false charges.

To promote the success of these defence organisations is, we will venture to suggest, one of the responsibilities that may be fairly placed on all those who have influence with the profession. Especially can a claim in this direction be pressed on the teachers in our medical schools, and more particularly on the teachers of medical jurisprudence. To these there come many opportunities of pointing out the legal risks and dangers which attend medical practice, and it thus falls to them to indicate how these risks and dangers may be most efficiently met. In our view, no medical student should leave his hospital or college without a strong recommendation to join a medical defence society.

